# Classical and Bayesian Inference for the Two-Parameter Rayleigh Distribution with Random Censored Data

**DOI:** 10.3390/e28030313

**Published:** 2026-03-10

**Authors:** Lanxi Zhang, Wenhao Gui, Zihan Zhao, Minghui Liu

**Affiliations:** School of Mathematics and Statistics, Beijing Jiaotong University, Beijing 100044, China; 23271053@bjtu.edu.cn (L.Z.); 23271112@bjtu.edu.cn (Z.Z.); 23271069@bjtu.edu.cn (M.L.)

**Keywords:** random censoring, survival analysis, Bayes estimation, reliability analysis

## Abstract

This study focuses on parameter estimation and reliability analysis for the two-parameter Rayleigh distribution under random censoring. It is shown that directly fitting the standard Rayleigh distribution can lead to substantial estimation errors, especially when the dataset contains a markedly high minimum value. To overcome the limitation of the conventional single-parameter Rayleigh distribution, which lacks a threshold parameter in practical applications, a two-parameter Rayleigh distribution model is proposed. The main research contents include the following: establishing a randomly censored data model; deriving classical inference methods based on maximum likelihood estimation along with several other classical estimation techniques; and constructing a Bayesian estimation framework. We also analyze several reliability and experimental characteristics by deriving their corresponding estimates. A Monte Carlo simulation study is carried out to assess the performance of the proposed estimators. Finally, the practicality and superiority of the two-parameter model are validated using real strength datasets. The results demonstrate that the two-parameter Rayleigh distribution can more accurately describe survival data with threshold characteristics and outperforms the single-parameter model in terms of model fit and reliability estimation.

## 1. Introduction

Survival analysis constitutes a methodology for examining expected lifetimes or event occurrence times, finding extensive applications across medicine, engineering, and social sciences. Within reliability theory, event occurrence times are also termed lifetime data or failure time data. Modeling and analyzing such lifetime data enables the assessment and prediction of reliability levels for products or systems, facilitating accurate inferences regarding statistical patterns over temporal dimensions. In the fields of reliability theory and survival analysis, parametric lifetime models like the exponential, Weibull, and gamma distributions offer a concise mathematical framework for characterizing patterns of “event occurrence times”. Advances in communications technology and the growing need to analyze underlying failure mechanisms now demand more precise and robust statistical distributions to model failure data.

First proposed in 1880 to describe the results of superimposed random vibrations, the Rayleigh distribution later gained prominence as a widely used lifetime model, driven by practical engineering needs in fields such as wireless communications. Numerous researchers have conducted in-depth studies on it within reliability theory. In practical scenarios, constraints from multiple factors often render obtaining “complete event times” difficult or prohibitively costly. As a direct consequence, a diverse array of censoring strategies are intentionally adopted in the course of lifetime experimental studies. The most conventional methods are Type I and Type II censoring. For instance, partial Bayes estimation has been developed to enhance inference under informative priors [1]. Within a progressively Type-II censored framework, methods have been proposed specifically for estimating parameters of the two-parameter Rayleigh model, improving efficiency in life-testing experiments [2]. Furthermore, robust explicit estimators are derived for the shifted Rayleigh distribution under Type-II censoring [3], addressing estimation stability in the presence of location parameters. These developments illustrate the continued methodological refinement of Rayleigh-based models in reliability analysis.Within such methodological frameworks, either the censoring duration or the quantity of censored samples is commonly set in advance in a deliberate manner. In actual observations, subjects are frequently lost or randomly removed before failure. This phenomenon of random removal at non-termination points is termed random censoring.

With randomly censored data, the authors of [4,5,6,7] extensively investigated various distributions, including the exponential, Rayleigh, and Burr Type XII distributions. In recent research efforts, the studies documented in [8,9] have specifically investigated Bayesian estimation methodologies tailored to generalized exponential and Weibull distributions under the scenario of random censoring. The authors of [10,11] have investigated estimation for both the Maxwell distribution and the generalized inverted exponential distribution in the context of randomly censored data. The authors of [12] further extended the scope of this field by investigating the parameter estimation problem of the exponential distribution with an introduced location parameter under random censoring. Recent research continues to expand into the study of other flexible models. For instance, the authors of [13] employed a combination of classical and Bayesian approaches to establish a comprehensive analytical framework for the randomly censored Kumaraswamy distribution.

For the conventional study of data with random censoring, it is assumed that failure times and censoring times both start from the zero point. However, in practice, most event times possess an inherent threshold, meaning events cannot occur before a minimum time (μ). Consequently, the assumptions of the standard Rayleigh distribution prove overly idealized and unrealistic in practical applications. For mathematical convenience, the censor time is also set to the same minimum threshold. Introducing a location parameter effectively corrects systematic fitting bias, enhances model flexibility and parameter interpretability, and indirectly improves inference accuracy. The introduction of a location parameter μ under random censoring is not a trivial extension—it brings new methodological challenges. Specifically, for the observed sample $y_{1},\ldots ,y_{n}$, the support of the distribution becomes data-dependent through the constraint $\mu <\min\{y_{1},\ldots ,y_{n}\}$, and the censoring mechanism interacts with the location parameter in ways that complicate both classical and Bayesian inference. Standard estimation procedures developed for the one-parameter case are no longer directly applicable, requiring the development of tailored inferential methods.

This paper addresses these challenges by developing comprehensive classical and Bayesian inference procedures for the two-parameter Rayleigh distribution under random censoring. We derive maximum likelihood estimators with closed-form expressions for μ, construct Fisher information matrices for asymptotic inference, and develop Gibbs sampling algorithms for Bayesian estimation under generalized entropy loss functions. Through extensive simulations and real data analysis, we demonstrate that the proposed methods not only extend the scope of existing censored-data methodologies but also provide more accurate and reliable estimates when the data exhibit a non-zero minimum lifetime.

The structure of the rest of this paper is set out below. Section 2 first introduces the basic definition of the two-parameter Rayleigh distribution, then constructs the mathematical model under random censoring. It specifies the distribution assumptions for failure and censoring times, along with the corresponding joint likelihood function of the model. Section 3 systematically expounds on classical parameter estimation methods. Section 4 moves on to the Bayesian theoretical framework, elaborating on the specific Bayesian estimation process constructed under the generalized entropy loss function with conjugate inverted-gamma prior distributions, the practical implementation of the Gibbs sampling algorithm for acquiring posterior sampling data, and the systematic construction of highest posterior density credible intervals by means of the Chen--Shao algorithm. Section 5 deals with the derivation and estimation of key reliability characteristics under the random-censoring model, including the mean time to system failure, hazard function, reliability function and expected time on test. Section 6 conducts extensive Monte Carlo simulation study to evaluate and compare the finite-sample performance of all the proposed estimators under various parameter configurations and prior settings. Section 7 provides a practical illustration using a carbon fiber strength dataset; goodness-of-fit tests and graphical comparisons demonstrate the superiority of the two-parameter Rayleigh model over its one-parameter counterpart, and the estimation methods developed in this paper are applied to obtain parameter estimates and reliability indices. Finally, Section 8 summarizes the work and discusses possible directions for future research. All numerical computations and simulations in this paper are performed using the statistical software R (Version 4.5.2).

## 2. The Model and Its Assumptions

The probability density function (PDF) pertaining to the two-parameter Rayleigh distribution can be expressed as follows:

$$f\left(x|\mu ,\sigma \right)=\frac{x-\mu }{\sigma^{2}}\exp\left(-\frac{{\left(x-\mu \right)}^{2}}{{2\sigma }^{2}}\right);\;0\leq \mu \leq x<\infty ,\sigma >0,$$

where σ denotes the scale parameter and μ the location parameter, which stands for the minimum lifetime of the studied units. This distribution is denoted by Rayleigh(μ,σ), and its corresponding cumulative distribution function (CDF) is defined as follows:

$$F\left(x|\mu ,\sigma \right)=1-\exp\left(-\frac{{\left(x-\mu \right)}^{2}}{{2\sigma }^{2}}\right);\;0\leq \mu \leq x<\infty ,\sigma >0.$$

**Figure 1:**
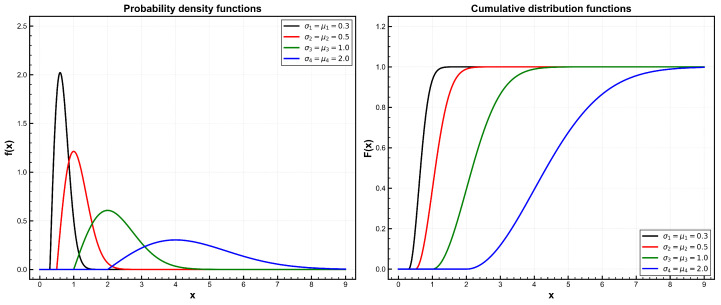
Left: probability density functions (PDFs) of the two-parameter Rayleigh distribution for σ=μ=0.3 (black), 0.5 (red), 1.0 (green), and 2.0 (blue); right: corresponding cumulative distribution functions (CDFs).

The two-parameter Rayleigh distribution offers greater flexibility than the standard Rayleigh distribution by incorporating a location parameter μ(μ≥0). This flexibility is particularly important in modeling scenarios where lifetime measurements do not originate from zero. Furthermore, the two-parameter Rayleigh distribution is invariably uni-modal and is characterized by an increasing hazard rate function. This renders it particularly well-suited for modeling the lifetime distributions of components subject to rapid aging over time. The probability density function and cumulative distribution function of this distribution are plotted in Figure 1.

Consider a test conducted on *n* specimens, where their individual lifetimes are denoted by $X_{1},X_{2},\ldots ,X_{n}$. These random variables follow an independent and identical distribution, with the corresponding PDF $f_{X}\left(x|\mu ,\sigma \right)$ and CDF $F_{X}\left(x|\mu ,\sigma \right)$.

Moreover, let $T_{1},T_{2},\ldots ,T_{n}$ represent the random censoring times for the aforementioned specimens, with their probability density function and cumulative distribution function given by $f_{T}\left(x|\mu ,\gamma \right)$ and $F_{T}\left(x|\mu ,\gamma \right)$, respectively. For mathematical tractability, we assume that the failure time $X_{i}$ and the censoring time $T_{i}$ share the same location parameter μ. This simplification, which has been commonly adopted in the literature on random censoring (e.g., [12]), enables a clean derivation of the joint likelihood and preserves the two-parameter Rayleigh structure for the marginal distribution of $Y_{i}$. We further assume that $X_{i}$ and $T_{i}$ are mutually independent for each *i*. It is important to note that in practice, only one of $X_{i}$ and $T_{i}$ can be observed for each test unit. Let the actual observed time be denoted as $Y_{i}=\min\left(X_{i},T_{i}\right)$ for i=1,2,…,n. In addition, an indicator variable $D_{i}$ is defined as follows:

$$D_{i}=\left\{\begin{array}{cc} 1 & \mathrm{if}\;X_{i}\leq T_{i}\\ 0 & \mathrm{if}\;X_{i}>T_{i} \end{array}\right.;\;i=1,2,\ldots ,n$$

Note that $D_{i}$ is a Bernoulli random variable whose probability mass function is defined as

$$P\left(D_{i}=d_{i}\right)=p^{d_{i}}{\left(1-p\right)}^{1-d_{i}};\;d_{i}=0,1,\;\mathrm{where}\;p=P\left(X_{i}\leq T_{i}\right).$$

The probability of failure prior to censoring is derived as follows:

$$\begin{array}{cc} p & =P\left[\mathrm{An}\;\mathrm{item}\;\mathrm{fails}\right]=P\left[D_{i}=1\right]=P\left[X_{i}\leq T_{i}\right]\\ \; & =\int_{\mu }^{\infty }f_{T}\left(t\right)F_{X}\left(t\right)\;dt\\ \; & =\int_{\mu }^{\infty }\frac{t-\mu }{\gamma^{2}}e^{-\frac{{\left(t-\mu \right)}^{2}}{2\gamma^{2}}}\left(1-e^{-\frac{{\left(t-\mu \right)}^{2}}{2\sigma^{2}}}\right)dt\\ \; & =\frac{\gamma^{2}}{\sigma^{2}+\gamma^{2}}. \end{array}$$

Substituting this result gives the explicit marginal distribution:

(1) $$P\left(D_{i}=d_{i}\right)={\left(\frac{\gamma^{2}}{\sigma^{2}+\gamma^{2}}\right)}^{d_{i}}{\left(\frac{\sigma^{2}}{\sigma^{2}+\gamma^{2}}\right)}^{1-d_{i}},\;d_{i}=0,1.$$

Note that the expression for the above probability does not contain μ. To see that $Y_{i}$ and $D_{i}$ are independent, consider the transformed variables $U_{i}={\left(X_{i}-\mu \right)}^{2}$ and $V_{i}={\left(T_{i}-\mu \right)}^{2}$. Under the assumptions that $X_{i}$ and $T_{i}$ are independent and share the same location parameter μ, it follows that $U_{i}$ and $V_{i}$ are independent and follow exponential distributions. Specifically, $U_{i}∼\mathrm{Exp}\left(1/\left(2\sigma^{2}\right)\right)$ and $V_{i}∼\mathrm{Exp}\left(1/\left(2\gamma^{2}\right)\right)$.

A classical result for exponential distributions (see [12]) states that, for two independent exponential variables, *U* and *V*, the event {U≤V} is independent of min(U,V). Applying this result to $U_{i}$ and $V_{i}$, we have that $\left\{U_{i}\leq V_{i}\right\}$ is independent of $\min(U_{i},V_{i})$. Since $\left\{U_{i}\leq V_{i}\right\}=\left\{X_{i}\leq T_{i}\right\}=\left\{D_{i}=1\right\}$, and $\min(U_{i},V_{i})$ is a one-to-one function of $Y_{i}=\min\left(X_{i},T_{i}\right)=\mu +\sqrt{\min(U_{i},V_{i})}$, it follows that $D_{i}$ and $Y_{i}$ are independent.

Therefore, the joint probability distribution of $\left(Y_{i},D_{i}\right)$ can be expressed as

(2) $$\begin{array}{cc} f_{Y,D}\left(y_{i},d_{i}|\mu ,\sigma ,\gamma \right) & ={\left\{f_{X}\left(y_{i}|\mu ,\sigma \right)\left(1-F_{T}\left(y_{i}|\mu ,\gamma \right)\right)\right\}}^{d_{i}}{\left\{f_{T}\left(y_{i}|\mu ,\gamma \right)\left(1-F_{X}\left(y_{i}|\mu ,\sigma \right)\right)\right\}}^{{1-d}_{i}}\\ & ={\left[\frac{y_{i}-\mu }{\sigma^{2}}\exp\left(-\frac{{\left(y_{i}-\mu \right)}^{2}}{{2\sigma }^{2}}\right)\exp\left(-\frac{{\left(y_{i}-\mu \right)}^{2}}{{2\gamma }^{2}}\right)\right]}^{d_{i}}\\ & \;\times {\left[\frac{y_{i}-\mu }{\gamma^{2}}\exp\left(-\frac{{\left(y_{i}-\mu \right)}^{2}}{{2\gamma }^{2}}\right)\exp\left(-\frac{{\left(y_{i}-\mu \right)}^{2}}{{2\sigma }^{2}}\right)\right]}^{{1-d}_{i}}\\ & ={\left(\frac{y_{i}-\mu }{\sigma^{2}}\right)}^{d_{i}}{\left(\frac{y_{i}-\mu }{\gamma^{2}}\right)}^{{1-d}_{i}}\exp\left(-\frac{{\left(y_{i}-\mu \right)}^{2}}{{2\sigma }^{2}}-\frac{{\left(y_{i}-\mu \right)}^{2}}{{2\gamma }^{2}}\right)\\ & ={\left(\frac{y_{i}-\mu }{\sigma^{2}}\right)}^{d_{i}}{\left(\frac{y_{i}-\mu }{\gamma^{2}}\right)}^{{1-d}_{i}}\exp\left(-\frac{{\left(y_{i}-\mu \right)}^{2}\beta }{2}\right) \end{array}$$

$$\begin{array}{c} y_{i}>\mu \geq 0,\;\sigma ,\gamma \geq 0,\;d_{i}=0,1,\;\beta :=\frac{1}{\sigma^{2}}+\frac{1}{\gamma^{2}}. \end{array}$$

where the parameter β is defined to simplify subsequent calculations.

The marginal distribution of $Y_{i}$ can be obtained by summing the joint distribution in (2) over $d_{i}=0,1$:

$$\begin{array}{cc} f_{Y}\left(y_{i}|\mu ,\sigma ,\gamma \right) & =\sum_{d_{i}=0}^{1}f_{Y,D}\left(y_{i},d_{i}\right)\\ & =\left(\frac{1}{\sigma^{2}}+\frac{1}{\gamma^{2}}\right)\left(y_{i}-\mu \right)\exp\left(-\frac{{\left(y_{i}-\mu \right)}^{2}\beta }{2}\right)\\ & =\beta \left(y_{i}-\mu \right)\exp\left(-\frac{{\left(y_{i}-\mu \right)}^{2}\beta }{2}\right),\;y_{i}>\mu , \end{array}$$

This is exactly the probability density function of a two-parameter Rayleigh distribution with location parameter μ and scale parameter $1/\sqrt{\beta }$, denoted as $\mathrm{Rayleigh}(\mu ,1/\sqrt{\beta })$.

The expected values for $Y_{i}$ and $D_{i}$ are derived as $E\left(Y_{i}\right)=\mu +\sqrt{\frac{\pi }{2\beta }}$ and $E\left(D_{i}\right)=\frac{\gamma^{2}}{\sigma^{2}+\gamma^{2}}=p$, in that order. On this basis, the collection $\left\{\left(y_{i},d_{i}\right);i=1,2,\ldots ,n\right\}$ constitutes a random censoring sample, for which the joint probability function is elaborated in (2), and the respective marginal distributions are formulated by the mathematical expressions presented in (1) and (2).

## 3. Classical Estimation Methods

Maximum likelihood estimation (MLE) is employed to infer the unknown model parameters, where the ensuing analytical procedures are conducted on the basis of a randomly censored sample designated as $\left(y,d\right)=\{\left(y_{i},d_{i}\right);i=1,2,\ldots ,n\}$. The likelihood function associated with this sample is formulated as follows:

(3) $$\begin{array}{cc} \; & L\left(\mu ,\sigma ,\gamma |y,d\right)=\prod_{i=1}^{n}f\left(y_{i},d_{i}\right)\\ \; & \;=\sigma^{-2\sum_{i=1}^{n}d_{i}}\gamma^{-2(n-\sum_{i=1}^{n}d_{i})}\left(\prod_{i=1}^{n}\left(y_{i}-\mu \right)\right)\exp\left[-\frac{\beta }{2}\sum_{i=1}^{n}{\left(y_{i}-\mu \right)}^{2}\right]\\ \; & \;y_{i}>\mu \geq 0;\;\sigma ,\gamma >0,\;\beta =\frac{1}{\sigma^{2}}+\frac{1}{\gamma^{2}}. \end{array}$$

Now, the log–likelihood function becomes

(4) $$\begin{array}{cc} \log L(\mu ,\sigma ,\gamma ) & =\log\left[\sigma^{-2\sum_{i=1}^{n}d_{i}}\gamma^{-2(n-\sum_{i=1}^{n}d_{i})}\left(\prod_{i=1}^{n}\left(y_{i}-\mu \right)\right)\exp\left[-\frac{\beta }{2}\sum_{i=1}^{n}{\left(y_{i}-\mu \right)}^{2}\right]\right]\\ & =-2\left(\sum_{i=1}^{n}d_{i}\right)\log\sigma -2\left(n-\sum_{i=1}^{n}d_{i}\right)\log\gamma +\sum_{i=1}^{n}\log\left(y_{i}-\mu \right)-\frac{\beta }{2}\sum_{i=1}^{n}{\left(y_{i}-\mu \right)}^{2} \end{array}$$

The likelihood is an increasing function of μ and μ is less than or equal to $\min\{y_{1},\ldots ,y_{n}\}$. Consequently, the MLE of the parameter μ is exactly given by $\hat{\mu }=\min\left\{y_{1},\ldots ,y_{n}\right\}$. Note that $\hat{\mu }=\min\left\{y_{1},\ldots ,y_{n}\right\}=y_{\left(1\right)}$ is a boundary estimator, as it coincides with the smallest observation, while the true parameter μ is strictly less than all realizations of $Y_{i}$. Consequently, this estimator exhibits positive bias in finite samples, with its expectation given in Proposition  1 as $E\left(\hat{\mu }\right)=\mu +\frac{1}{\sqrt{n}}\sqrt{\frac{\pi }{2\beta }}$. The bias decreases as the sample size *n* increases, confirming the consistency of $\hat{\mu }$.

Taking $w_{1}=\sum_{i=1}^{n}d_{i}$, $w_{2}=\sum_{i=1}^{n}{\left(y_{i}-\hat{\mu }\right)}^{2}$. The MLEs corresponding to parameters σ and γ can be derived by numerically solving these two associated equations $\frac{\partial \log L(\mu ,\sigma ,\gamma )}{\partial \sigma }=0$ and $\frac{\partial \log L(\mu ,\sigma ,\gamma )}{\partial \gamma }=0$ as

(5) $${\hat{\sigma }}^{2}=\frac{\sum_{i=1}^{n}{\left(y_{i}-\hat{\mu }\right)}^{2}}{2\sum_{i=1}^{n}d_{i}}=\frac{w_{2}}{2w_{1}}\;and\;{\hat{\gamma }}^{2}=\frac{\sum_{i=1}^{n}{\left(y_{i}-\hat{\mu }\right)}^{2}}{2(n-\sum_{i=1}^{n}d_{i})}=\frac{w_{2}}{2(n-w_{1})}$$

### 3.1. Variance in the Maximum Likelihood Estimator

**Proposition** **1.** *Let $y_{1},y_{2},\ldots ,y_{n}$ denote independent and identically distributed random variables that share a common probability density function*

(6) $$f\left(y_{i}|\mu ,\beta \right)=\beta \left(y_{i}-\mu \right)\exp\left(-\frac{{\left(y_{i}-\mu \right)}^{2}\beta }{2}\right);\;y_{i}>\mu \geq 0,\;\;\beta =\frac{1}{\sigma^{2}}+\frac{1}{\gamma^{2}}.$$

*Then the minimum order statistic $y_{\left(1\right)}=\min\left\{y_{1},y_{2},\ldots ,y_{n}\right\}$ follows a two-parameter Rayleigh distribution with location parameter μ and scale parameter $1/\sqrt{n\beta }$, i.e.,*

(7) $$y_{\left(1\right)}∼\mathrm{Rayleigh}\;\left(\mu ,\;\frac{1}{\sqrt{n\beta }}\right).$$

**Proof.**  The CDF corresponding to (6) is

(8) $$F\left(y_{i}|\mu ,\beta \right)=1-\exp\left(-\frac{{\left(y_{i}-\mu \right)}^{2}\beta }{2}\right);\;y_{i}>\mu \geq 0.$$

For the minimum of *n* independent random variables, we have

$$\begin{array}{cc} F_{y_{\left(1\right)}}\left(y_{i}\right) & =1-P\;\left(y_{\left(1\right)}>y_{i}\right)\\ & =1-P\;\left(y_{1}>y_{i},y_{2}>y_{i},\ldots ,y_{n}>y_{i}\right)\\ & =1-\prod_{i=1}^{n}P\;\left(y_{i}>y_{i}\right)\\ & =1-{\left[1-F_{y}\left(y_{i}\right)\right]}^{n}. \end{array}$$

Substituting (8) gives

$$\begin{array}{cc} F_{y_{\left(1\right)}}\left(y_{i}\right) & =1-{\left[1-\left(1-\exp\;\left(-\frac{{\left(y_{i}-\mu \right)}^{2}\beta }{2}\right)\right)\right]}^{n}\\ & =1-{\left[\exp\;\left(-\frac{{\left(y_{i}-\mu \right)}^{2}\beta }{2}\right)\right]}^{n}\\ & =1-\exp\;\left(-\frac{n{\left(y_{i}-\mu \right)}^{2}\beta }{2}\right). \end{array}$$

Rewriting the exponent

(9) $$F_{y_{\left(1\right)}}\left(y_{i}\right)=1-\exp\;\left(-\frac{{\left(y_{i}-\mu \right)}^{2}\left(n\beta \right)}{2}\right);\;y_{i}>\mu \geq 0.$$

Expression (9) coincides with the cumulative distribution function of a two-parameter Rayleigh distribution having location μ and scale $1/\sqrt{n\beta }$. Differentiating (9) with respect to $y_{i}$ gives the corresponding probability density function

$$\begin{array}{cc} f_{y_{\left(1\right)}}\left(y_{i}\right) & =\frac{d}{dy}F_{y_{\left(1\right)}}\left(y_{i}\right)\\ & =n\beta \left(y_{i}-\mu \right)\exp\;\left(-\frac{{\left(y_{i}-\mu \right)}^{2}n\beta }{2}\right);\;y_{i}>\mu \geq 0. \end{array}$$

This density matches precisely the form of a Rayleigh density with parameters μ and $1/\sqrt{n\beta }$.

Consequently, it follows that $y_{\left(1\right)}∼\mathrm{Rayleigh}\;\left(\mu ,\;1/\sqrt{n\beta }\right)$, which thereby completes the theoretical proof.    □

Since $y_{\left(1\right)}$ follows $\mathrm{Rayleigh}(\mu ,1/\sqrt{n\beta })$, the mean and variance in the $\hat{\mu }$ are

$$E\left(\hat{\mu }\right)=E\left(y_{\left(1\right)}\right)=\mu +\frac{1}{\sqrt{n}}\sqrt{\frac{\pi }{2\beta }}\;\mathrm{and}\;\hat{V}\left(\hat{\mu }\right)=\frac{4-\pi }{2n\hat{\beta }}=\frac{\left(4-\pi \right)w_{2}}{4n^{2}}.$$

Note that $\hat{\mu }=y_{\left(1\right)}$ is a consistent but biased estimator of μ. By estimating μ with the sample minimum, the original three-parameter estimation problem reduces to a two-parameter problem involving only σ and γ, simplifying subsequent inference.

The second-order partial derivatives, needed for the observed Fisher information matrix, are

$$\frac{\partial^{2}\log L\left(\mu ,\sigma ,\gamma \right)}{\partial \sigma^{2}}=\frac{2w_{1}}{\sigma^{2}}-\frac{3w_{2}}{\sigma^{4}}$$

$$\frac{\partial^{2}\log L\left(\mu ,\sigma ,\gamma \right)}{\partial \gamma^{2}}=\frac{2(n-w_{1})}{\gamma^{2}}-\frac{3w_{2}}{\gamma^{4}}$$

$$\frac{\partial^{2}\log L\left(\mu ,\sigma ,\gamma \right)}{\partial \sigma \partial \gamma }=0=\frac{\partial^{2}\log L\left(\mu ,\sigma ,\gamma \right)}{\partial \gamma \partial \sigma }.$$

It is worth acknowledging that the maximum likelihood estimator of μ lies on the boundary of the parameter space, which may affect the regularity conditions underlying the Fisher information matrix. However, the MLE of μ is super-consistent and converges at rate *n*, which mitigates its impact on the asymptotic distribution of $\hat{\sigma }$ and $\hat{\gamma }$. Nevertheless, the Fisher information matrix derived below focuses on the scale parameters σ and γ, which are interior points, and the resulting asymptotic intervals are commonly used in practice as approximations.

From (1), we have

$$E\left(w_{1}\right)=E\left(\sum_{i=1}^{n}D_{i}\right)=np=\frac{n\gamma^{2}}{\sigma^{2}+\gamma^{2}}.$$

For the Rayleigh distribution, it can be shown that

$$E\left(w_{2}\right)=E\left[\sum_{i=1}^{n}{\left(y_{i}-\hat{\mu }\right)}^{2}\right]=\frac{2\left(n-1\right)\sigma^{2}\gamma^{2}}{\sigma^{2}+\gamma^{2}}.$$

Now, the Fisher information matrix for (σ,γ) can be derived as

(10) $$\begin{array}{cc} I(\sigma ,\gamma ) & =-E\left(\begin{array}{cc} \frac{\partial^{2}\log L\left(\mu ,\sigma ,\gamma \right)}{\partial \sigma^{2}} & \frac{\partial^{2}\log L\left(\mu ,\sigma ,\gamma \right)}{\partial \sigma \partial \gamma }\\ \frac{\partial^{2}\log L\left(\mu ,\sigma ,\gamma \right)}{\partial \gamma \partial \sigma } & \frac{\partial^{2}\log L\left(\mu ,\sigma ,\gamma \right)}{\partial \gamma^{2}} \end{array}\right)\\ \; & =\left(\begin{array}{cc} \frac{4n\gamma^{2}}{\sigma^{2}\left(\sigma^{2}+\gamma^{2}\right)} & 0\\ 0 & \frac{4n\sigma^{2}}{\gamma^{2}\left(\sigma^{2}+\gamma^{2}\right)} \end{array}\right) \end{array}$$

Since the Fisher information matrix I(σ,γ) is diagonal here, it can be conveniently inverted by simply inverting each element along its main diagonal. The diagonal elements of the inverse information matrix $I^{-1}\left(\sigma ,\gamma \right)$ provide the asymptotic variance estimators for the maximum likelihood estimators (MLEs). Specifically, these variance estimates are given by

(11) $$V\left(\hat{\sigma }\right)=\frac{\sigma^{2}\left(\sigma^{2}+\gamma^{2}\right)}{4n\gamma^{2}}\;\mathrm{and}\;V\left(\hat{\gamma }\right)=\frac{\gamma^{2}\left(\sigma^{2}+\gamma^{2}\right)}{4n\sigma^{2}}$$

These variance expressions depend on the unknown parameters σ and γ. For practical application, usable estimates of the variances are obtained by replacing σ and γ with their corresponding MLEs. The estimated variances for the estimators $\hat{\sigma }$ and $\hat{\gamma }$ are subsequently derived as follows:

(12) $$V\left(\hat{\sigma }\right)=\frac{{\hat{\sigma }}^{2}\left({\hat{\sigma }}^{2}+{\hat{\gamma }}^{2}\right)}{4n{\hat{\gamma }}^{2}}=\frac{w_{2}}{8w_{1}^{2}}$$

(13) $$V\left(\hat{\gamma }\right)=\frac{{\hat{\gamma }}^{2}\left({\hat{\sigma }}^{2}+{\hat{\gamma }}^{2}\right)}{4n{\hat{\sigma }}^{2}}=\frac{w_{2}}{8{\left(n-w_{1}\right)}^{2}}$$

### 3.2. Asymptotic Confidence Intervals

Given that $y_{\left(1\right)}$ follows a $\mathrm{Rayleigh}(\mu ,1/\sqrt{n\beta })$ distribution, the two-sided equal-tailed confidence interval of (1−α)×100% for the parameter μ can be readily deduced in accordance with the method presented in reference [12]. Let

$$w_{2}^{*}=\sum_{i=1}^{n}{\left(y_{i}-y_{\left(1\right)}\right)}^{2},$$

Then, the confidence interval (CI) of μ is

$$\left[y_{\left(1\right)}-\sqrt{\frac{w_{2}^{*}}{2n(n-1)}F_{2,2(n-1)}\left(1-\alpha /2\right)},\;y_{\left(1\right)}-\sqrt{\frac{w_{2}^{*}}{2n(n-1)}F_{2,2(n-1)}\left(\alpha /2\right)}\right],$$

where $F_{2,2(n-1)}\left(\alpha /2\right)$ denotes the upper α/2×100% critical value of Snedecor’s *F*-distribution with numerator and denominator degrees of freedom equal to 2 and 2(n−1), respectively. Herein, (1−α) serves as the confidence coefficient, with the parameter α satisfying the constraint that 0<α<1.

This interval construction assumes that $y_{\left(1\right)}$ is sufficient and that the data are exactly follow the two-parameter Rayleigh distribution. Under model misspecification, the actual coverage probability may differ from the nominal level. In such cases, bootstrap methods offer a more robust alternative for interval estimation of μ.

For the scale parameters σ and γ, the asymptotic sampling distribution associated with the MLEs $\left(\hat{\sigma },\hat{\gamma }\right)$ follows a bivariate normal distribution denoted as $\mathrm{BND}\left(\left(\sigma ,\gamma \right),I^{-1}\left(\sigma ,\gamma \right)\right)$. On this basis, we are able to derive the CI for the parameters (σ,γ) by means of the normal approximation method. The two-sided 100×(1−α)% CI corresponding to σ and γ can be expressed as

$${\mathrm{CI}}_{\sigma }:\;\hat{\sigma }\pm z_{1-\alpha /2}\sqrt{\hat{\mathrm{var}}\left(\hat{\sigma }\right)},$$

$${\mathrm{CI}}_{\gamma }:\;\hat{\gamma }\pm z_{1-\alpha /2}\sqrt{\hat{\mathrm{var}}\left(\hat{\gamma }\right)},$$

Here, $z_{1-\alpha /2}$ refers to the upper (1−α/2)th quantile of the standard normal distribution. It should be noted that these confidence intervals are based on asymptotic normal approximations, which may not be accurate for small or moderately sized samples, especially under heavy censoring. In practice, alternative methods such as bootstrap confidence intervals could be considered to potentially improve coverage accuracy in such scenarios.

In Section 6, we carry out a Monte Carlo simulation experiment to calculate the upper and lower confidence bounds of the parameters as well as the average lengths of their confidence intervals by using simulated samples, and further obtain the corresponding coverage probabilities for these intervals accordingly.

### 3.3. Method of Moments

Upon equating sample moments with the corresponding population moments, the relevant estimates of the model parameters are obtainable via this moment-matching approach. For the random variables *D* and *Y*, we define their sample counterparts based on the observed data as follows: $\bar{d}=\frac{1}{n}\sum_{i=1}^{n}d_{i}$, $\;\bar{y}=\frac{1}{n}\sum_{i=1}^{n}y_{i}$, $\;s_{y}^{2}=\frac{1}{n}\sum_{i=1}^{n}{\left(y_{i}-\bar{y}\right)}^{2}$ and population moments $E\left(D\right)=\frac{\gamma^{2}}{\sigma^{2}+\gamma^{2}}$,   $E\left(Y\right)=\mu +\sqrt{\frac{\pi \sigma^{2}\gamma^{2}}{2(\sigma^{2}+\gamma^{2})}}$,   $V\left(Y\right)=\frac{4-\pi }{2}\cdot \frac{\sigma^{2}\gamma^{2}}{\sigma^{2}+\gamma^{2}}$. By equating the respective moments to one another, we thus obtain

$$\bar{d}=\frac{\gamma^{2}}{\sigma^{2}+\gamma^{2}};\;\bar{y}=\mu +\sqrt{\frac{\pi \sigma^{2}\gamma^{2}}{2(\sigma^{2}+\gamma^{2})}};\;s_{y}^{2}=\frac{4-\pi }{2}\cdot \frac{\sigma^{2}\gamma^{2}}{\sigma^{2}+\gamma^{2}}$$

We can derive the moment estimators corresponding to each parameter as

$$\hat{\mu }=\bar{y}-s_{y}\sqrt{\frac{\pi }{4-\pi }};\;{\hat{\sigma }}^{2}=\frac{2s_{y}^{2}}{\left(4-\pi \right)\bar{d}};\;{\hat{\gamma }}^{2}=\frac{2s_{y}^{2}}{\left(4-\pi \right)\left(1-\bar{d}\right)}$$

It can be observed that these moment estimators provide unique solutions for μ, $\sigma^{2}$, and $\gamma^{2}$ since the sample moments satisfy $0<\bar{d}<1$ and $s_{y}^{2}>0$ in practice. This indicates that the parameters are identifiable from the chosen moments.

### 3.4. Least-Squares Estimates

$Y_{\left(1\right)},Y_{\left(2\right)},\ldots ,Y_{\left(n\right)}$ denote the order statistics from the marginal distribution of *Y*, where *Y* obeys a two-parameter Rayleigh distribution with location parameter μ and scale parameter $1/\sqrt{\beta }$. The parameter β is defined as $\beta =\frac{1}{\sigma^{2}}+\frac{1}{\gamma^{2}}$. The cumulative distribution function (CDF) corresponding to the random variable *Y* is expressed as

$$F_{Y}\left(y\right)=1-\exp\left[-\frac{\beta }{2}{\left(y-\mu \right)}^{2}\right];\;y>\mu \geq 0.$$

The expected value of $F_{Y}\left(Y_{\left(i\right)}\right)$ is approximately

$$E\left[F_{Y}\left(Y_{\left(i\right)}\right)\right]=\frac{i}{n+1};\;i=1,2,\ldots ,n.$$

The least-squares estimates of the parameters μ, σ, and γ can be obtained by minimizing the following objective function

$$Q_{1}=\sum_{i=1}^{n}{\left[F_{Y}\left(Y_{\left(i\right)}\right)-E\left[F_{Y}\left(Y_{\left(i\right)}\right)\right]\right]}^{2}=\sum_{i=1}^{n}{\left[1-\exp\left(-\frac{\beta }{2}{\left(Y_{\left(i\right)}-\mu \right)}^{2}\right)-\frac{i}{n+1}\right]}^{2};\;i=1,2,\ldots ,n.$$

To account for the heterogeneity in the variances of $F_{Y}\left(Y_{\left(i\right)}\right)$, we consider the weighted least-squares method. The variance in $F_{Y}\left(Y_{\left(i\right)}\right)$ is approximately

$$V\left[F_{Y}\left(Y_{\left(i\right)}\right)\right]=\frac{i(n-i+1)}{{\left(n+1\right)}^{2}\left(n+2\right)};\;i=1,2,\ldots ,n.$$

Taking the weights as $k_{i}=1/V\left[F_{Y}\left(Y_{\left(i\right)}\right)\right]$, the weighted least-squares estimates are acquired through the minimization of

$$Q_{2}=\sum_{i=1}^{n}k_{i}{\left[F_{Y}\left(Y_{\left(i\right)}\right)-E\left[F_{Y}\left(Y_{\left(i\right)}\right)\right]\right]}^{2}=\sum_{i=1}^{n}\frac{{\left(n+1\right)}^{2}\left(n+2\right)}{i(n-i+1)}{\left[1-\exp\left(-\frac{\beta }{2}{\left(Y_{\left(i\right)}-\mu \right)}^{2}\right)-\frac{i}{n+1}\right]}^{2}.$$

The minimization is carried out for μ, σ, and γ, where $\beta =\frac{1}{\sigma^{2}}+\frac{1}{\gamma^{2}}$.

## 4. Bayesian Estimation

We proceed to develop a Bayesian inferential framework for the unknown parameters associated with the two-parameter Rayleigh distribution, where the whole analytical process is based on randomly censored data. On this basis, we derive the Bayes estimators for the aforementioned parameters under the generalized entropy loss function (GELF), as well as the associated highest posterior density (HPD)-credible intervals corresponding to each parameter. We then advance the Bayesian inference method for σ and γ with randomly censored data as the research basis; specifically, we impose the prior assumption that $\sigma^{2}$ and $\gamma^{2}$, respectively, follow conjugate inverse gamma distributions with hyperparameters $\left(a_{1},b_{1}\right)$ and $\left(a_{2},b_{2}\right)$, and the corresponding PDF are expressed as

(14) $$g_{1}\left(\sigma^{2}|a_{1},b_{1}\right)\propto \frac{1}{{\left(\sigma^{2}\right)}^{a_{1}+1}}e^{-b_{1}/\sigma^{2}};\;\sigma^{2}>0,a_{1},b_{1}>0.$$

(15) $$g_{2}\left(\gamma^{2}|a_{2},b_{2}\right)\propto \frac{1}{{\left(\gamma^{2}\right)}^{a_{2}+1}}e^{-b_{2}/\gamma^{2}};\;\gamma^{2}>0,a_{2},b_{2}>0.$$

For the location parameter μ, we introduce the subsequent improper uniform prior distribution

(16) $$g_{3}\left(\mu \right)\propto \frac{1}{c};\;0\leq \mu <y_{\left(1\right)},\;c>0.$$

The parameters μ, $\sigma^{2}$, and $\gamma^{2}$ are assumed to be independent priori.

The joint posterior distribution is

$$\begin{array}{cc} \pi (\mu ,\sigma^{2},\gamma^{2}|y,d)\; & =\frac{L\left(y,d|\mu ,\sigma^{2},\gamma^{2}\right)g_{1}\left(\sigma^{2}|a_{1},b_{1}\right)g_{2}\left(\gamma^{2}|a_{2},b_{2}\right)g_{3}\left(\mu \right)}{\;\int \int \int \;L\left(y,d|\mu ,\sigma^{2},\gamma^{2}\right)g_{1}\left(\sigma^{2}|a_{1},b_{1}\right)g_{2}\left(\gamma^{2}|a_{2},b_{2}\right)g_{3}\left(\mu \right)\;d\mu \;d\sigma^{2}\;d\gamma^{2}} \end{array}$$

(17) $$\begin{array}{c} =\frac{\frac{1}{{\left(\sigma^{2}\right)}^{a_{1}+w_{1}+1}}e^{-\left(b_{1}+\sum_{i=1}^{n}y_{i}-n\mu \right)/\sigma^{2}}\frac{1}{{\left(\gamma^{2}\right)}^{a_{2}+n-w_{1}+1}}e^{-\left(b_{2}+\sum_{i=1}^{n}y_{i}-n\mu \right)/\gamma^{2}}}{\int_{-\infty }^{y_{\left(1\right)}}\int_{0}^{\infty }\int_{0}^{\infty }\frac{1}{{\left(\sigma^{2}\right)}^{a_{1}+w_{1}+1}}e^{-\left(b_{1}+\sum_{i=1}^{n}y_{i}-n\mu \right)/\sigma^{2}}\frac{1}{{\left(\gamma^{2}\right)}^{a_{2}+n-w_{1}+1}}e^{-\left(b_{2}+\sum_{i=1}^{n}y_{i}-n\mu \right)/\gamma^{2}}\;d\gamma^{2}\;d\sigma^{2}\;d\mu }; \end{array}$$

where $w_{1}=\sum_{i=1}^{n}d_{i}$.

This joint posterior density function does not admit an explicit closed form. Therefore, to compute the Bayes estimates, one may employ a numerical integration procedure based on the posterior distribution given in (17) under an appropriate loss function. Alternatively, the importance sampling algorithm can be employed to acquire simulation-consistent Bayesian estimators and construct the corresponding HPD-credible intervals, in accordance with the methodological approach introduced by [14].

To implement the Gibbs sampling procedure, we employ a technique analogous to that described by [15]. Setting $b_{1}=b_{2}=b$, we take the posterior as

$$\pi \left(\mu ,\sigma^{2},\gamma^{2}|y,d\right)\propto L\left(y,d|\mu ,\sigma^{2},\gamma^{2}\right)\cdot g_{1}\left(\sigma^{2}\right)\cdot g_{2}\left(\gamma^{2}\right)\cdot g_{3}\left(\mu \right),$$

It follows that the joint posterior distribution can be written in the form of

$$\pi \left(\mu ,\sigma^{2},\gamma^{2}|y,d\right)=\frac{f_{1}\left(\sigma^{2}|\mu ,y,d\right)\;f_{2}\left(\gamma^{2}|\mu ,y,d\right)\;f_{3}\left(\mu |y,d\right)}{\underset{\mu <y_{\left(1\right)}}{\;\int \int \int \;}f_{1}\left(\sigma^{2}|\mu ,y,d\right)\;f_{2}\left(\gamma^{2}|\mu ,y,d\right)\;f_{3}\left(\mu |y,d\right)\;d\mu \;d\sigma^{2}\;d\gamma^{2}}$$

where,

$$\begin{array}{cc} f_{1}\left(\sigma^{2}|\mu ,y,d\right) & \propto {\left(\sigma^{2}\right)}^{-(a_{1}+w_{1}+1)}\exp\left[-\frac{1}{\sigma^{2}}\left(b+\frac{1}{2}S\left(\mu \right)\right)\right],\\ f_{2}\left(\gamma^{2}|\mu ,y,d\right) & \propto {\left(\gamma^{2}\right)}^{-(a_{2}+n-w_{1}+1)}\exp\left[-\frac{1}{\gamma^{2}}\left(b+\frac{1}{2}S\left(\mu \right)\right)\right],\\ f_{3}\left(\mu |y,d\right) & \propto \left(\prod_{i=1}^{n}\left(y_{i}-\mu \right)\right)\exp\left[-\frac{\beta }{2}S\left(\mu \right)\right]I_{\left(-\infty ,y_{\left(1\right)}\right)}\left(\mu \right),\\ \mathrm{with}\;S(\mu ) & =2\sum_{i=1}^{n}y_{i}-2n\mu =2n\left(\bar{y}-\mu \right). \end{array}$$

We note here that $f_{1}\left(\sigma^{2}|\mu ,y,d\right)$ and $f_{2}\left(\gamma^{2}|\mu ,y,d\right)$ correspond to inverse gamma density functions with a common scale parameter $\left(b+\frac{1}{2}\sum_{i=1}^{n}{\left(y_{i}-\mu \right)}^{2}\right)$ but different shape parameters $\left(a_{1}+w_{1}\right)$ and $\left(a_{2}+n-w_{1}\right)$, respectively.

The density function $f_{3}\left(\mu |y,d\right)$ is a valid proper density function. Its cumulative distribution function (CDF) can be derived as

$$F_{3}\left(\mu \right)=\frac{{\left(b+\frac{1}{2}\sum_{i=1}^{n}{\left(y_{i}-y_{\left(1\right)}\right)}^{2}\right)}^{\;a_{1}+a_{2}+n-1}}{{\left(b+\frac{1}{2}\sum_{i=1}^{n}{\left(y_{i}-\mu \right)}^{2}\right)}^{\;a_{1}+a_{2}+n-1}};\;0\leq \mu <y_{\left(1\right)}.$$

This CDF is readily invertible, allowing for straightforward generation of random samples for μ via the probability integral transform. Specifically, for a uniform random variate u∼U(0,1), we can obtain a sample of μ by solving

(18) $$\mu =F_{3}^{-1}\left(u\right)=y_{\left(1\right)}-\sqrt{\frac{2}{n}\left[\frac{b+\frac{1}{2}\sum_{i=1}^{n}{\left(y_{i}-y_{\left(1\right)}\right)}^{2}}{u^{1/(a_{1}+a_{2}+n-1)}}-b\right]}.$$

Since the prior for μ is now defined on the finite interval $\left[0,y_{\left(1\right)}\right)$, it is a proper prior. Together with the proper priors for $\sigma^{2}$ and $\gamma^{2}$, the joint posterior distribution $\pi (\mu ,\sigma^{2},\gamma^{2}|y,d)$ is guaranteed to be proper, ensuring the validity of Bayesian inference even for small sample sizes. This invertibility facilitates the implementation of the Gibbs sampling procedure.

### 4.1. Bayes Estimates Under GELF

For Bayesian estimation, we employ the generalized entropy loss function (GELF). Although this loss function was introduced earlier [16], it remains relevant in modern reliability analysis, as demonstrated in recent Bayesian studies on lifetime distributions under various censoring schemes [13,17]. The generalized entropy loss function is defined as follows:

(19) $$L\left(\theta ,\theta^{*}\right)=\left[{\left(\frac{\theta^{*}}{\theta }\right)}^{\delta }-\delta \log\left(\frac{\theta^{*}}{\theta }\right)-1\right];\;\delta \neq 0.$$

Here, $\theta^{*}$ is an estimate of θ. For δ<0, underestimation incurs a more severe penalty than overestimation, whereas for δ>0, the situation is reversed, with overestimation being penalized more heavily. In particular, δ=1 corresponds to the entropy loss function (ELF), δ=2 corresponds to the squared error loss function (SELF), and δ=−1 corresponds to the precautionary loss function (PLF). These values represent different degrees of asymmetry and allow us to comprehensively assess the impact of the loss function on Bayesian estimates.

Within the Bayesian estimation framework, we select the $\theta^{*}$ value that minimizes the risk function $r\left(\theta ,\theta^{*}\right)=E\left[L(\theta ,\theta^{*})|y,d\right]$. Deriving the Bayes estimator of θ involves computing the derivative of $r(\theta ,\theta^{*})$ with respect to $\theta^{*}$ and equating the derived expression to zero. The Bayes estimator for θ and its related risk function are formulated as

(20) $$\theta^{*}={\left\{E\left(\theta^{-\delta }|y,d\right)\right\}}^{-1/\delta }$$

(21) $$r\left(\theta ,\theta^{*}\right)=E\left[\log\left(\theta^{\delta }\right)|y,d\right]+\log\left[E\left(\theta^{-\delta }|y,d\right)\right]=E\left[\log\left(\theta^{\delta }\right)|y,d\right]-\log{\left(\theta^{*}\right)}^{\delta }.$$

We can also characterize Bayesian estimation of σ and γ under the GELF.

Although the improper uniform prior $g_{3}\left(\mu \right)\propto 1$ is used for the location parameter μ, the resulting joint posterior distribution $\pi (\mu ,\sigma^{2},\gamma^{2}|y,d)$ remains proper. This can be seen by noting that the likelihood function in (3) contains the factor $\prod_{i=1}^{n}\left(y_{i}-\mu \right)$, which is bounded on the interval $\left[0,y_{\left(1\right)}\right)$ since $0\leq y_{i}-\mu \leq y_{i}-0<\infty$. The exponential term $\exp\left[-\frac{\beta }{2}\sum_{i=1}^{n}{\left(y_{i}-\mu \right)}^{2}\right]$ is also bounded on this interval. Hence, the likelihood, viewed as a function of μ, is bounded on the truncated support $\left[0,y_{\left(1\right)}\right)$.

Moreover, the constraint $\mu <y_{\left(1\right)}$ imposed by the observed data truncates the support of μ to the finite interval $\left[0,y_{\left(1\right)}\right)$. Over this compact set, the bounded likelihood combined with the proper inverse gamma priors for $\sigma^{2}$ and $\gamma^{2}$ ensures that the joint posterior is integrable. More formally,

$$\int_{0}^{y_{\left(1\right)}}\int_{0}^{\infty }\int_{0}^{\infty }L\left(\mu ,\sigma^{2},\gamma^{2}|y,d\right)\;g_{1}\left(\sigma^{2}\right)\;g_{2}\left(\gamma^{2}\right)\;g_{3}\left(\mu \right)\;d\sigma^{2}\;d\gamma^{2}\;d\mu <\infty ,$$

because:

- $L(\mu ,\sigma^{2},\gamma^{2}|y,d)$ is bounded in μ on $\left[0,y_{\left(1\right)}\right)$;
- $g_{1}\left(\sigma^{2}\right)$ and $g_{2}\left(\gamma^{2}\right)$ are proper inverse gamma densities;
- The improper prior $g_{3}\left(\mu \right)\propto 1$ integrates to a finite constant $y_{\left(1\right)}$ over the finite interval $\left[0,y_{\left(1\right)}\right)$.

Consequently, the joint posterior is well-defined and valid for Bayesian inference.

### 4.2. Gibbs Sampling Procedure

The Gibbs sampling algorithm (Algorithm 1), which is used to generate samples from the joint posterior distribution $\pi (\mu ,\sigma^{2},\gamma^{2}|y,d)$, is implemented as follows:
**Algorithm 1** Gibbs Sampling for Bayesian Estimation under GELF.**Require:** Initial values $\mu^{\left(0\right)},\sigma^{2(0)},\gamma^{2(0)}$; hyperparameters $a_{1},a_{2},b$; total iterations *M*; burn-in period *B*; loss function parameter δ
 1:**for** t=1 **to** *M* **do** 2:    **Sample** $\sigma^{2(t)}$**:** $\sigma^{2(t)}∼\mathrm{Inv}\text{-}\mathrm{Gamma}\left(a_{1}+w_{1},\;b+\frac{1}{2}\sum_{i=1}^{n}{\left(y_{i}-\mu^{\left(t-1\right)}\right)}^{2}\right)$ 3:    **Sample** $\gamma^{2(t)}$**:** $\gamma^{2(t)}∼\mathrm{Inv}\text{-}\mathrm{Gamma}\left(a_{2}+n-w_{1},\;b+\frac{1}{2}\sum_{i=1}^{n}{\left(y_{i}-\mu^{\left(t-1\right)}\right)}^{2}\right)$ 4:    **Sample** $\mu^{\left(t\right)}$ **(using inverse CDF method):** 5:    Generate u∼U(0,1) 6:    Compute $\mu^{\left(t\right)}=F_{3}^{-1}\left(u\right)$ 7:**end for** 8:**Burn-in:** Discard first *B* samples: ${\left\{\left(\mu^{\left(j\right)},\sigma^{\left(j\right)},\gamma^{\left(j\right)}\right)\right\}}_{j=1}^{B}$ 9:**Bayesian estimation under GELF:**10:$\mu^{*}={\left[\frac{1}{M-B}\sum_{j=B+1}^{M}{\left(\mu^{\left(j\right)}\right)}^{-\delta }\right]}^{-1/\delta }$11:$\sigma^{*}={\left[\frac{1}{M-B}\sum_{j=B+1}^{M}{\left(\sigma^{\left(j\right)}\right)}^{-\delta }\right]}^{-1/\delta }$12:$\gamma^{*}={\left[\frac{1}{M-B}\sum_{j=B+1}^{M}{\left(\gamma^{\left(j\right)}\right)}^{-\delta }\right]}^{-1/\delta }$13:**Return:** $\mu^{*},\sigma^{*},\gamma^{*}$


When no prior information is available, we can employ non-informative prior distributions for the relevant parameters. For the scale parameters $\sigma^{2}$ and $\gamma^{2}$, this corresponds to setting $a_{1}=a_{2}=b=0$ in the inverted gamma priors. For the location parameter μ, we retain the improper uniform prior $g_{3}\left(\mu \right)\propto 1$. Under these non-informative priors, the posterior distribution is dominated by the likelihood.

Convergence of the Gibbs sampler was assessed using trace plots and the Gelman–Rubin diagnostic. We ran three parallel chains with over-dispersed starting values, each for *M* = 20,000 iterations, discarding the first B=5000 iterations as burn-in. In all cases, $\hat{R}$ values were below 1.1, indicating that convergence was achieved. The effective sample sizes (ESSs) for all parameters exceeded 1000, ensuring reliable posterior inference.

### 4.3. HPD-Credible Intervals and Chen–Shao Algorithm

A 100(1−α)% highest posterior density (HPD) credible interval is defined to be an interval that fulfills the two subsequent criteria: it contains a posterior probability mass of (1−α), and the posterior density at every interior point within the interval exceeds that at all exterior points outside the interval. As a result, the HPD interval is the shortest credible interval achievable for a specified probability level of (1−α). Based on these basic properties, ref. [14] proposed a computational procedure for deriving the HPD-credible interval corresponding to the parameter σ.

The Chen–Shao algorithm [14] is given in Algorithm 2.
**Algorithm 2** Finding the HPD-credible interval.**Require:** MCMC total iterations *M*, burn-in period *B*, significance level α
 1:**Step 1: Prepare effective posterior samples** 2:Retain the effective samples after burn-in: $\left\{\sigma_{j}^{2},j=B+1,B+2,\ldots ,M\right\}$, giving an effective sample size N=M−B. 3:Sort the effective sample set in ascending order to obtain the order statistics:$$\sigma_{\left(1\right)}^{2}\leq \sigma_{\left(2\right)}^{2}\leq \ldots \leq \sigma_{\left(N\right)}^{2}$$ 4:**Step 2: Generate candidate HPD intervals.** 5:Set K=⌊(1−α)N⌋. 6:**for** j=1 **to** N−K **do** 7:    $I_{j}=\left[\sigma_{\left(j\right)}^{2},\sigma_{\left(j+K\right)}^{2}\right]$, $w_{j}=\sigma_{\left(j+K\right)}^{2}-\sigma_{\left(j\right)}^{2}$ 8:**end for** 9:**Step 3: Identify the HPD interval (minimum width).**10:$I_{\mathrm{HPD}}=\arg\min_{j=1,\ldots ,N-K}w_{j}=\left[\sigma_{\mathrm{low}}^{2},\sigma_{\mathrm{up}}^{2}\right]$11:**Step 4: Output the result.**12:Return the 100(1−α)% HPD-credible interval for $\sigma^{2}$: $\left[\sigma_{\mathrm{low}}^{2},\sigma_{\mathrm{up}}^{2}\right]$


The same procedure can be applied to obtain HPD-credible intervals for $\gamma^{2}$ and μ. Specifically, after obtaining the posterior samples ${\left\{\gamma_{j}^{2}\right\}}_{j=B+1}^{M}$ and ${\left\{\mu_{j}\right\}}_{j=B+1}^{M}$ from the Gibbs sampling algorithm, we sort each set of samples in ascending order and apply Steps 2–3 of Algorithm 2 to find the shortest intervals that contain the desired posterior probability. This yields the 100(1−α)% HPD-credible intervals for $\gamma^{2}$ and μ, respectively.

## 5. Reliability and Experimental Characteristics Estimation

Within the present analytical framework, the two-parameter Rayleigh distribution parameterized by (μ,σ) is employed to model lifetime data, while its reliability metrics and experimental behavior are examined using the corresponding estimators derived under a randomly censored sampling scheme.

A key practical consideration is whether a life test can be finished within a predefined time interval. As experimental costs depend directly on the test duration, this information is key to selecting a suitable sampling strategy and determining the sample size for life test specimens.

In random censoring model, the observed time $Y_{i}=\min\left(X_{i},T_{i}\right)$ follows a two-parameter Rayleigh distribution $\mathrm{Rayleigh}(\mu ,1/\sqrt{\beta })$, where $\beta =\frac{1}{\sigma^{2}}+\frac{1}{\gamma^{2}}$. The completion time of the experiment is determined by the maximum observed value, i.e., $Y_{\left(n\right)}=\max\left(Y_{1},Y_{2},\ldots ,Y_{n}\right)$.

The cumulative distribution function of $Y_{\left(n\right)}$ is

(22) $$F_{Y_{\left(n\right)}}\left(y\right)=P\left[Y_{\left(n\right)}\leq y\right]={\left[1-\exp\left(-\frac{\beta }{2}{\left(y-\mu \right)}^{2}\right)\right]}^{n},\;y>\mu \geq 0.$$

The expected time on test (ETT) is defined as the expectation of $Y_{\left(n\right)}$

(23) $$\mathrm{ETT}=E\left[Y_{\left(n\right)}\right]=\mu +\int_{0}^{\infty }\left[1-F_{Y_{\left(n\right)}}\left(\mu +t\right)\right]dt.$$

Substituting (22) into (23) yields

(24) $$\mathrm{ETT}=\mu +\int_{0}^{\infty }\left[1-{\left(1-e^{-\beta t^{2}/2}\right)}^{n}\right]dt.$$

For the Rayleigh distribution, this integral lacks a straightforward closed-form expression yet can be solved via numerical methods. In practice, the observed time on test (OBTT), which is $Y_{\left(n\right)}$ itself, serves as a natural estimator of ETT.

For the complete data (no censoring) scenario, the test time is determined by the maximum failure time $X_{\left(n\right)}$, and its expected value is

(25) $${\mathrm{ETT}}_{\mathrm{complete}}=E\left[X_{\left(n\right)}\right]=\mu +\int_{0}^{\infty }\left[1-{\left(1-e^{-t^{2}/\left(2\sigma^{2}\right)}\right)}^{n}\right]dt.$$

where $\left\{\left(\mu_{j},\sigma_{j},\gamma_{j}\right);\;j=1,2,\ldots ,M\right\}$ are the posterior samples obtained through Gibbs sampling, and ETT(μ,σ,γ) is computed using Equation (24).

Since the expected time on test does not have a closed-form expression for the Rayleigh distribution, both $\hat{\mathrm{ETT}}$ and ${\mathrm{ETT}}^{*}$ require numerical integration. For each parameter set (μ,σ,γ), we compute the integral in Equation (24) using adaptive numerical integration methods. In the Bayesian framework, this computation is performed for each posterior sample $\left(\mu_{j},\sigma_{j},\gamma_{j}\right)$, and the results are then combined according to Table 1.

For the numerical computation of the expected time on test (ETT), we employed adaptive numerical integration as implemented in the R integrate function. The integration was performed over the interval [0,μ+10max(σ,γ)], which was verified to capture the essential support of the distribution. The relative tolerance was set to $10^{-4}$ (for estimation) and $10^{-6}$ (for true value computation), with subdivisions up to 1000 to ensure accuracy. Based on our implementation, the computational cost of a single ETT evaluation is approximately 0.002 s on a standard desktop computer. In the simulation study with 10 parameter combinations, three sample sizes (n=20,50,100), and $N_{\mathrm{sim}}=5000$ replications per configuration, the total time for ETT estimation was approximately 0.2 min, which was negligible compared to the overall computational burden.

## 6. Simulation Studies

A Monte Carlo simulation experiment is carried out to assess and compare the performance of the various estimators proposed previously. Specifically, to facilitate a direct comparison between classical and Bayesian inference methods, we generate randomly censored samples across a range of values for parameters μ, σ, and γ. For each generated sample, we calculate the MLEs along with their respective confidence intervals. Besides the Bayes estimators derived under the generalized entropy loss function, along with the associated HPD-credible intervals corresponding to such estimators.

Before detailing the simulation procedure, we clarify the selection of hyperparameters for informative priors. They are chosen so that the prior means equal the true parameter values (i.e., $\sigma^{2}=b/\left(a_{1}-1\right)$ and $\gamma^{2}=b/\left(a_{2}-1\right)$), ensuring that the prior information is centered correctly. This allows for a meaningful evaluation of the Bayesian estimators’ performance when prior information is accurate.

We now outline the simulation procedure step-by-step as Algorithm 3.
**Algorithm 3** Monte Carlo simulation procedure.**Require:** Conjugate inverted gamma priors with hyperparameters $\left(a_{1},b\right)$ and $\left(a_{2},b\right)$, sample sizes n=20,50,100 to investigate the finite-sample performance under small, moderate, and large sample scenarios, number of simulations N=5000, and mission time *s*.**Ensure:** Average values and mean squared errors of parameter estimates, reliability characteristics, ETT and OBTT1:Select values of hyperparameters $\left(a_{1},b\right)$ and $\left(a_{2},b\right)$ for informative priors, and set $a_{1}=a_{2}=b=0$ for non-informative priors.2:Determine distinct parameter values equal to the means of the prior distributions, such that $\sigma^{2}=b/\left(a_{1}-1\right)$ and $\gamma^{2}=b/\left(a_{2}-1\right)$ hold, respectively.3:Compute the true MTSF value using $\mathrm{MTSF}=\mu +\sigma \sqrt{\pi /2}$, where μ is the location parameter for minimum lifetime. Next, the remaining reliability and experimental performance indicators are calculated at a mission time of s=μ+MTSF/2.4:Produce a random sample y of sample size n=20,50,100 from $\mathrm{Rayleigh}(\mu ,1/\sqrt{\beta })$, where $\beta =\frac{1}{\sigma^{2}}+\frac{1}{\gamma^{2}}$ and PDF is given in (2), for different parameter values. Similarly, generate d from the pmf in (1).5:For the comparative study, various estimators and corresponding intervals are computed for different parameter and hyperparameter values, following the procedures outlined in Section 3 and Section 4.6:Obtain the corresponding estimates of all relevant reliability characteristics, including ETT and OBTT.7:Repeat Steps (4) to (6) a total of N=5000 iterations for distinct parameter value combinations. For each estimator derived from Steps (5) to (6), calculate its corresponding AV and MSE.

To compare the performance of the Bayesian estimation method and the classical estimation method, we try different parameter values and hyperparameter values, with the primary simulation results shown in Table 2, Table 3, Table 4, Table 5, Table 6 and Table 7.

Based on the comprehensive analysis of the results presented in the aforementioned tables, it can be concluded that

- Maximum likelihood estimation: MLE exhibits systematic bias that decreases with increasing sample size. The bias is more pronounced in small samples, while both bias and MSE improve significantly as sample size increases, confirming the consistency property of MLE but also highlighting its limitations in small-sample scenarios.
- Least-squares and weighted least-squares: Both LS and WLS methods provide reasonable parameter estimates across all sample sizes. For small samples (n=20), LS and WLS show notable bias, particularly for the scale parameters σ and γ, with MSE values considerably larger than those of MLE and Bayesian methods. However, as sample size increases to n=50 and n=100, the performance of both methods improves substantially, with bias and MSE decreasing markedly. WLS generally yields slightly lower MSE than LS for most parameter combinations, particularly for larger parameter values, confirming the benefit of incorporating variance heterogeneity in the estimation procedure.
- Expected time on test (ETT): The ETT estimates exhibit similar patterns to the parameter estimates, with bias and MSE decreasing as sample size increases. For n=20, the ETT estimates show considerable bias, especially for combinations with larger parameter values. However, the bias is substantially reduced for n=50 and n=100, with MSE decreasing by approximately 50% to 70% compared to the small sample case, indicating that reliable ETT estimation requires moderate to large sample sizes. The numerical integration procedure proved to be both accurate and computationally efficient, with negligible impact on overall simulation time.
- Comparison of Bayesian methods: The SELF estimator outperforms both MLE and non-informative priors across all sample sizes, with its advantage being most pronounced in small samples. Although this advantage diminishes as sample size increases, it remains present, indicating that informative priors provide crucial stability when data are scarce.
- Effect of prior information: Non-informative priors perform reasonably well but yield larger MSEs than SELF, with the difference being more substantial in small samples. As sample size increases, the gap narrows considerably. This demonstrates that the advantage of Bayesian methods stems primarily from the informative prior structure, and this benefit is most prominent when sample information is limited.
- Interval estimation: The asymptotic confidence intervals for μ, σ, and γ show coverage probabilities that improve with increasing sample size. For n=20, the coverage probabilities are below the nominal 95% level for all parameters, particularly for μ. As sample size increases to n=50 and n=100, coverage probabilities approach the nominal level for μ and show improvement for σ and γ, although some undercoverage remains. Interval lengths decrease consistently with increasing sample size, as expected. These results suggest that caution is warranted when interpreting confidence intervals in small samples, especially for the scale parameters.
- Sample size effects: The most dramatic improvement across all estimators occurs between small and moderate sample sizes, with diminishing returns thereafter. This suggests that a moderate sample size provides a reasonable balance between estimation accuracy and experimental cost in practical applications.
- Practical recommendations: Based on the systematic comparison across sample sizes, we recommend prioritizing Bayesian methods such as SELF for small samples; for moderate samples, SELF remains the preferred choice while non-informative priors and LS/WLS methods serve as viable alternatives; for large samples, all methods are acceptable, though Bayesian methods still offer some efficiency gains. For ETT estimation, moderate to large sample sizes (n≥50) are recommended to achieve reliable results. The interval estimation method for μ becomes reliable for n≥50, but caution is advised when interpreting σ and γ intervals, particularly in small samples.

To assess the impact of the loss function parameter δ on the Bayesian estimates, we compare the results obtained under three different loss functions: SELF (δ=−2), ELF (δ=−1), and PLF (δ=1). Table 6 presents the parameter estimates for selected parameter combinations with sample size n=50.

The results in Table 8 show that the parameter estimates are relatively stable across different loss functions. The differences between SELF, ELF, and PLF are generally small, with slightly larger variations observed for PLF in some cases (e.g., for σ in the (5,5,3) combination). This indicates that the Bayesian estimation procedure is robust to the choice of the loss function parameter δ within a reasonable range.

To provide a structured comparison, Table 9 presents the relative efficiency (RE = MSE_SELF/MSE_MLE) for selected parameter combinations. Values less than 1 indicate that SELF outperforms MLE.

The results show that all RE values are below 1, confirming the consistent superiority of Bayesian methods across all scenarios. The improvement is most pronounced for σ and γ estimation, with RE values as low as 0.04, and becomes more evident as sample size increases.

## 7. Real Data Example Analysis

To further validate the practical applicability of the proposed methodological framework, an illustrative empirical analysis is conducted herein by utilizing a carbon fiber strength dataset first published in the work of [15]. This dataset documents the strength values of individual carbon fibers as well as impregnated 1000-carbon fiber tows, with the full set of experimental data tabulated in Table 10. In line with the goodness-of-fit verification tests and the scaled total time on test (TTT) transformation method put forward by [15], the Rayleigh distribution is verified to be a suitable probabilistic model for characterizing the statistical properties of this carbon fiber strength dataset.

This analysis aims to show the superior precision of the location-scale Rayleigh model over the standard scale-only version. We consider model fitting for the dataset using: (i) a two-parameter Rayleigh model (σ, μ) and (ii) a one-parameter Rayleigh model (σ), with parameters estimated by maximum likelihood. The goodness of fit for these models is then evaluated based on several metrics: negative log–likelihood, Akaike Information Criterion (AIC), Bayesian Information Criterion (BIC), the Kolmogorov–Smirnov (K–S) statistic, and the empirical cumulative distribution function (ECDF).

For the K-S test, the null hypothesis $H_{0}$ is that the observed randomly censored data follow the specified Rayleigh distribution. A small *p*-value (typically <0.05) leads to the rejection of $H_{0}$, suggesting that the distributional assumption may not be appropriate.

The formula for calculating AIC is as follows:

AIC=2k−2log(L),

where *k* denotes the number of parameters incorporated into the statistical model, and *L* represents the maximum likelihood value associated with the estimated model.

The BIC is mathematically expressed as

BIC=klog(n)−2log(L).

where *k* and *L* retain their definitions from the AIC formula, and *n* refers to the total number of observations in the dataset (Table 11 and Table 12).

**Figure 2:**
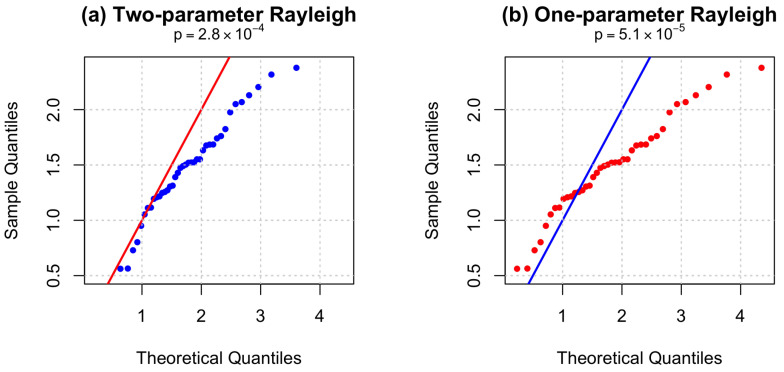
Q-Q plots for (**a**) two-parameter and (**b**) one-parameter Rayleigh distributions based on failure times.

It should be noted, however, that although the two-parameter Rayleigh distribution provides a better fit than its one-parameter counterpart, the Kolmogorov–Smirnov test yields *p*-values below 0.05 for both models, indicating that neither fully captures the empirical distribution of the carbon fiber strength data at conventional significance levels. This suggests that while the two-parameter model offers a substantial improvement, there may still be room for further refinement, such as considering more flexible distributions or mixture models. Q-Q plots are provided in Figure 2, which visually confirm the improved fit of the two-parameter model. Although both models show significant deviation from the 45-degree line (K-S test p<0.05), indicating imperfect fit, the two-parameter model demonstrates noticeably better alignment, particularly in the upper tail. This superior fit is consistent with its lower AIC and BIC values in Table 12, confirming that the inclusion of a location parameter improves model performance even when the overall fit is not ideal.

Based on all goodness-of-fit criteria, we find that the two-parameter Rayleigh distribution achieves substantially lower values of negative log–likelihood, AIC, and BIC compared to the one-parameter Rayleigh distribution. Therefore, the two-parameter Rayleigh distribution exhibits markedly superior fitting performance to the one-parameter Rayleigh distribution.

**Figure 3:**
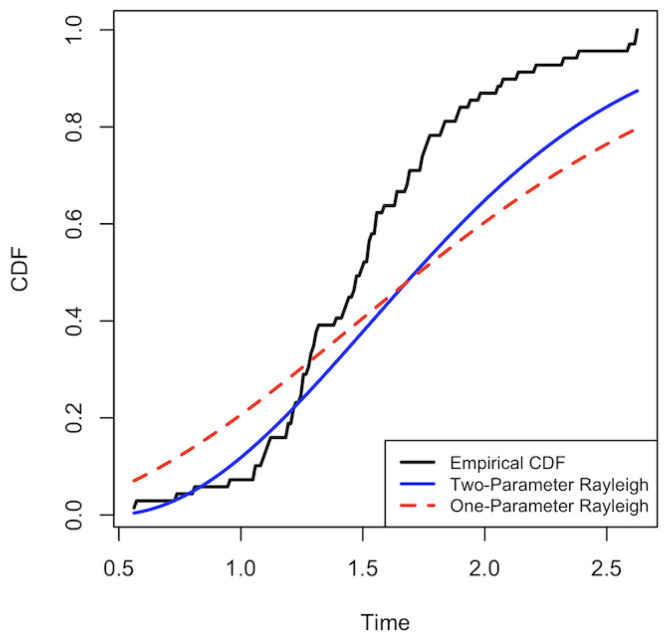
CDF curves for the strength data. ECDF of the strength data (black), fitted CDFs from the two-parameter (red) and one-parameter (blue) Rayleigh distributions.

For the graphical comparison, we present the cumulative distribution curves and examine their respective behaviors. As shown in Figure 3, the CDF curve of the two-parameter Rayleigh distribution lies closer to the empirical CDF than that of the one-parameter model. Consequently, we conclude that fitting this real dataset with the two-parameter Rayleigh distribution yields a considerably better fit than the one-parameter Rayleigh distribution, which only includes a scale parameter.

## 8. Conclusions

Randomly censored data are commonly encountered in both survival analysis and reliability theory. Numerous statistical distributions have been extensively utilized in prior research to develop estimation procedures under this data framework. This study introduces location parameter modeling into the analysis of randomly censored data following the Rayleigh distribution, thereby establishing a novel analytical framework for lifetime processes characterized by monotonically increasing failure rates. The inclusion of a location parameter is expected to influence estimates derived from data. The core objective of this study is to integrate this location parameter into models for both lifetime and censoring time distributions.

This study investigates a diverse set of estimation approaches for the location and scale parameters within the framework of the two-parameter Rayleigh distribution. A number of classical estimation methods are explored herein. In addition, Bayesian estimators corresponding to the parameters, as well as their CIs and HPD-credible intervals, are constructed under multiple loss functions. Given the theoretical challenges associated with comparing different estimation methods, a Monte Carlo simulation study is conducted to assess the performance of the various parameter estimators and their associated reliability characteristics. Comparisons among these estimators are mainly based on two key metrics: bias and mean squared error. Owing to the integration of supplementary prior information, Bayesian estimators can generate more accurate results than maximum likelihood estimators (MLEs) in certain cases, especially when the parameter values are large and the sample sizes are small. To demonstrate the practical applicability of the estimation methodologies proposed in this study, a real-world case analysis is presented using carbon fiber strength data. The empirical results show that the fitting performance of the two-parameter Rayleigh distribution is significantly superior to that of its one-parameter counterpart.

Future research could relax the assumption that failure and censoring times share the same location parameter, allowing these two processes to have different location parameters. Furthermore, the modeling framework of the two-parameter Rayleigh distribution could be extended to more complex survival analysis scenarios. For instance, covariates could be introduced to establish regression models, thereby analyzing the influence of various factors on lifetime distributions. Alternatively, the distribution could be integrated with structures such as competing risks or frailty models to handle more complex failure mechanisms and heterogeneous data.

## Figures and Tables

**Table 1:** Maximum likelihood estimates and Bayesian estimates under GELF of reliability and experimental characteristics.

Maximum Likelihood Estimate	Bayesian Estimate Under GELF
$\hat{\mathrm{MTSF}}=\hat{\mu }+\hat{\sigma }\sqrt{\frac{\pi }{2}}$	${\mathrm{MTSF}}^{*}={\left[\frac{1}{M-B}\sum_{j=B+1}^{M}{\left(\mu_{j}+\sigma_{j}\sqrt{\frac{\pi }{2}}\right)}^{-\delta }\right]}^{-1/\delta }$
$\hat{h}\left(s\right)=\frac{s-\hat{\mu }}{{\hat{\sigma }}^{2}};\;\hat{\mu }<s<\infty$	$h^{*}\left(s\right)={\left[\frac{1}{M-B}\sum_{j=B+1}^{M}{\left(\frac{s-\mu_{j}}{\sigma_{j}^{2}}\right)}^{-\delta }\right]}^{-1/\delta }$
$\hat{R}\left(s\right)=\exp\left(-\frac{{\left(s-\hat{\mu }\right)}^{2}}{2{\hat{\sigma }}^{2}}\right);\;\hat{\mu }<s<\infty$	$R^{*}\left(s\right)={\left[\frac{1}{M-B}\sum_{j=B+1}^{M}\exp\left(\frac{\delta {\left(s-\mu_{j}\right)}^{2}}{2\sigma_{j}^{2}}\right)\right]}^{-1/\delta }$
$\hat{p}=\frac{{\hat{\gamma }}^{2}}{{\hat{\sigma }}^{2}+{\hat{\gamma }}^{2}}$	$p^{*}={\left[\frac{1}{M-B}\sum_{j=B+1}^{M}{\left(\frac{\gamma_{j}^{2}}{\sigma_{j}^{2}+\gamma_{j}^{2}}\right)}^{-\delta }\right]}^{-1/\delta }$
$\hat{\mathrm{ETT}}=\hat{\mu }+\int_{0}^{\infty }\left[1-{\left(1-e^{-\hat{\beta }t^{2}/2}\right)}^{n}\right]dt$	
where $\hat{\beta }=\frac{1}{{\hat{\sigma }}^{2}}+\frac{1}{{\hat{\gamma }}^{2}}$	${\mathrm{ETT}}^{*}={\left[\frac{1}{M-B}\sum_{j=B+1}^{M}{\left(\mathrm{ETT}(\mu_{j},\sigma_{j},\gamma_{j})\right)}^{-\delta }\right]}^{-1/\delta }$

**Table 2:** MLEs for the parameters with different sample sizes.

	Parameters			$\hat{\mu }$		$\hat{\sigma }$		$\hat{\gamma }$	
n	μ	σ	γ	AV	MSE	AV	MSE	AV	MSE
20	0.5	1	1	0.6995	0.0509	0.5754	0.1963	0.5752	0.1955
	1	1	1	1.1932	0.0480	0.5738	0.1958	0.5764	0.1944
	2	1	1	2.1957	0.0489	0.5700	0.1991	0.5808	0.1921
	5	1	2	5.2454	0.0758	0.7363	0.0850	0.6997	1.7607
	0.5	2	3	0.9641	0.2750	1.3652	0.4595	1.3455	2.8740
	1	2	3	1.4663	0.2802	1.3631	0.4648	1.3275	2.9357
	2	3	2	2.4656	0.2759	1.3332	2.9064	1.3651	0.4634
	5	5	3	5.7418	0.7049	2.0655	8.9963	2.0962	0.9557
	9	1	1	9.1973	0.0495	0.5757	0.1954	0.5777	0.1935
50	0.5	1	1	0.6235	0.0195	0.6245	0.1472	0.6238	0.1475
	1	1	1	1.1243	0.0196	0.6276	0.1451	0.6222	0.1490
	2	1	1	2.1254	0.0199	0.6248	0.1468	0.6204	0.1502
	5	1	2	5.1565	0.0312	0.7967	0.0475	0.7843	1.5002
	0.5	2	3	0.7875	0.1071	1.4734	0.3053	1.4657	2.4067
	1	2	3	1.3022	0.1146	1.4718	0.3065	1.4584	2.4290
	2	3	2	2.2954	0.1110	1.4526	2.4406	1.4693	0.3061
	5	5	3	5.4545	0.2626	2.2623	7.6487	2.2849	0.5751
	9	1	1	9.1256	0.0199	0.6263	0.1457	0.6248	0.1468
100	0.5	1	1	0.5913	0.0106	0.6486	0.1267	0.6492	0.1261
	1	1	1	1.0900	0.0104	0.6486	0.1265	0.6517	0.1245
	2	1	1	2.0875	0.0096	0.6545	0.1227	0.6514	0.1248
	5	1	2	5.1155	0.0169	0.8218	0.0348	0.8150	1.4155
	0.5	2	3	0.7064	0.0541	1.5313	0.2333	1.5274	2.1952
	1	2	3	1.2080	0.0539	1.5319	0.2319	1.5315	2.1810
	2	3	2	2.2106	0.0571	1.5317	2.1833	1.5260	0.2379
	5	5	3	5.3275	0.1349	2.3504	7.0922	2.3616	0.4388
	9	1	1	9.0893	0.0101	0.6499	0.1259	0.6511	0.1249

**Table 3:** Expected time on test (ETT) estimates under different methods.

	Parameters			ETT Estimates			Bias			MSE		
n	μ	σ	γ	$\hat{\mu }$	$\hat{\sigma }$	$\hat{\gamma }$	μ	σ	γ	μ	σ	γ
20	0.5	1	1	0.7022	0.8366	0.8376	0.2022	−0.1634	−0.1624	0.0513	0.0554	0.0559
	1	1	1	1.1967	0.8434	0.8420	0.1967	−0.1566	−0.1580	0.0504	0.0563	0.0569
	2	1	1	2.1920	0.8393	0.8389	0.1920	−0.1607	−0.1611	0.0471	0.0500	0.0500
	5	1	2	5.2475	0.8270	1.7951	0.2475	−0.1730	−0.2049	0.0784	0.0499	0.4074
	0.5	2	3	0.9602	1.6533	2.6078	0.4602	−0.3467	−0.3922	0.2701	0.1914	0.5927
	1	2	3	1.4582	1.6611	2.6465	0.4582	−0.3389	−0.3535	0.2674	0.1948	0.7783
	2	3	2	2.4662	2.5636	1.6689	0.4662	−0.4364	−0.3311	0.2780	0.6209	0.1871
	5	5	3	5.7158	4.3634	2.4981	0.7158	−0.6366	−0.5019	0.6563	1.9941	0.4312
	9	1	1	9.2004	0.8414	0.8398	0.2004	−0.1586	−0.1602	0.0512	0.0493	0.0560
50	0.5	1	1	0.6243	0.8959	0.8950	0.1243	−0.1041	−0.1050	0.0198	0.0225	0.0218
	1	1	1	1.1251	0.9000	0.9029	0.1251	−0.1000	−0.0971	0.0196	0.0223	0.0209
	2	1	1	2.1312	0.8938	0.8864	0.1312	−0.1062	−0.1136	0.0216	0.0230	0.0243
	5	1	2	5.1546	0.8927	1.8532	0.1546	−0.1073	−0.1468	0.0299	0.0195	0.1381
	0.5	2	3	0.7939	1.7679	2.6988	0.2939	−0.2321	−0.3012	0.1111	0.0908	0.2441
	1	2	3	1.2927	1.7852	2.7110	0.2927	−0.2148	−0.2890	0.1091	0.0838	0.2663
	2	3	2	2.2916	2.7112	1.7843	0.2916	−0.2888	−0.2157	0.1100	0.2704	0.0901
	5	5	3	5.4569	4.5279	2.6759	0.4569	−0.4721	−0.3241	0.2612	0.7908	0.1803
	9	1	1	9.1256	0.8900	0.8950	0.1256	−0.1100	−0.1050	0.0198	0.0222	0.0223
100	0.5	1	1	0.5873	0.9287	0.9316	0.0873	−0.0713	−0.0684	0.0097	0.0108	0.0107
	1	1	1	1.0873	0.9243	0.9239	0.0873	−0.0757	−0.0761	0.0099	0.0116	0.0120
	2	1	1	2.0938	0.9237	0.9203	0.0938	−0.0763	−0.0797	0.0112	0.0119	0.0127
	5	1	2	5.1123	0.9212	1.8657	0.1123	−0.0788	−0.1343	0.0161	0.0103	0.0735
	0.5	2	3	0.7098	1.8432	2.7859	0.2098	−0.1568	−0.2141	0.0559	0.0451	0.1269
	1	2	3	1.2126	1.8390	2.7963	0.2126	−0.1610	−0.2037	0.0566	0.0428	0.1215
	2	3	2	2.2051	2.8055	1.8442	0.2051	−0.1945	−0.1558	0.0527	0.1153	0.0424
	5	5	3	5.3326	4.6549	2.7580	0.3326	−0.3451	−0.2420	0.1396	0.3575	0.0990
	9	1	1	9.0893	0.9271	0.9232	0.0893	−0.0729	−0.0768	0.0102	0.0118	0.0119

**Table 4:** Bayesian estimates under the SELF for the parameters.

	Parameters			$\hat{\mu }$		$\hat{\sigma }$		$\hat{\gamma }$	
n	μ	σ	γ	AV	MSE	AV	MSE	AV	MSE
20	0.5	1	1	0.4672	0.0129	1.0130	0.0295	1.0212	0.0279
	1	1	1	0.9797	0.0201	1.0086	0.0569	1.0352	0.0359
	2	1	1	1.9624	0.0166	1.0631	0.0400	1.0228	0.0391
	5	1	2	4.9684	0.0193	0.9985	0.0204	1.8485	0.1865
	0.5	2	3	0.4651	0.0988	1.9474	0.1025	2.8835	0.4526
	1	2	3	0.9135	0.1076	2.0019	0.1210	2.8936	0.4886
	2	3	2	2.0401	0.0956	2.8111	0.5761	1.8882	0.1325
	5	5	3	4.9570	0.4902	4.5388	2.0185	2.8110	0.3373
	9	1	1	8.9407	0.0160	1.0297	0.0239	1.0804	0.0462
50	0.5	1	1	0.4828	0.0062	1.0180	0.0131	1.0141	0.0101
	1	1	1	0.9801	0.0047	1.0359	0.0122	1.0209	0.0141
	2	1	1	1.9681	0.0070	1.0274	0.0114	1.0161	0.0127
	5	1	2	4.9705	0.0112	1.0127	0.0070	2.0336	0.1010
	0.5	2	3	0.4541	0.0296	1.9945	0.0417	2.9320	0.1761
	1	2	3	0.9647	0.0322	1.9894	0.0428	2.9833	0.1940
	2	3	2	1.9500	0.0329	2.9872	0.1560	2.0293	0.0496
	5	5	3	4.9072	0.0705	4.9659	0.6095	3.0175	0.0960
	9	1	1	8.9813	0.0060	1.0151	0.0099	1.0327	0.0129
100	0.5	1	1	0.4848	0.0028	1.0125	0.0068	0.9957	0.0050
	1	1	1	0.9960	0.0030	1.0063	0.0065	1.0060	0.0084
	2	1	1	1.9824	0.0027	1.0147	0.0082	1.0088	0.0072
	5	1	2	4.9853	0.0055	1.0034	0.0053	1.9798	0.0606
	0.5	2	3	0.4772	0.0139	1.9915	0.0221	3.0067	0.0917
	1	2	3	0.9635	0.0126	2.0087	0.0198	3.0246	0.0861
	2	3	2	1.9459	0.0135	2.9983	0.0770	2.0129	0.0250
	5	5	3	4.9618	0.0393	5.0538	0.3835	2.9973	0.0523
	9	1	1	8.9863	0.0024	1.0109	0.0048	1.0170	0.0062

**Table 5:** Least-squares (LS) and weighted least-squares (WLS) estimates for the parameters.

	Parameters			LS						WLS					
n	μ	σ	γ	$\hat{\mu }$		$\hat{\sigma }$		$\hat{\gamma }$		$\hat{\mu }$		$\hat{\sigma }$		$\hat{\gamma }$	
				AV	MSE	AV	MSE	AV	MSE	AV	MSE	AV	MSE	AV	MSE
20	0.5	1	1	0.7022	0.0513	0.5598	0.2073	0.5724	0.2106	0.7022	0.0513	0.5205	0.2414	0.5498	0.2308
	1	1	1	1.1967	0.0504	0.5659	0.2019	0.5902	0.2397	1.1967	0.0504	0.5239	0.2381	0.5681	0.2642
	2	1	1	2.1920	0.0471	0.5647	0.2021	0.5813	0.2071	2.1920	0.0471	0.5222	0.2386	0.5574	0.2275
	5	1	2	5.2475	0.0784	0.7418	0.0957	0.7925	1.6669	5.2475	0.0784	0.6914	0.1255	0.7896	1.6810
	0.5	2	3	0.9602	0.2701	1.5988	0.6696	2.2024	5.2736	0.9602	0.2701	1.5118	0.7729	2.2186	5.4581
	1	2	3	1.4582	0.2674	1.6118	0.6086	2.0867	4.3347	1.4582	0.2674	1.5244	0.7174	2.1255	4.6352
	2	3	2	2.4662	0.2780	1.6352	2.3650	1.7572	0.9852	2.4662	0.2780	1.5451	2.6403	1.7174	1.0824
	5	5	3	5.7158	0.6563	2.9477	7.6179	3.1405	4.5675	5.7158	0.6563	2.8171	8.2234	3.0896	4.8803
	9	1	1	9.2004	0.0512	0.5665	0.2018	0.5901	0.1997	9.2004	0.0512	0.5241	0.2385	0.5651	0.2198
50	0.5	1	1	0.6243	0.0198	0.6052	0.1641	0.6104	0.1663	0.6243	0.0198	0.5615	0.1999	0.5823	0.1873
	1	1	1	1.1251	0.0196	0.6090	0.1613	0.6155	0.1633	1.1251	0.0196	0.5649	0.1962	0.5862	0.1854
	2	1	1	2.1312	0.0216	0.6034	0.1654	0.6131	0.1642	2.1312	0.0216	0.5538	0.2056	0.5763	0.1923
	5	1	2	5.1546	0.0299	0.8497	0.0443	0.9254	1.3316	5.1546	0.0299	0.7761	0.0720	0.9109	1.3798
	0.5	2	3	0.7939	0.1111	2.0367	0.4976	2.2205	2.1552	0.7939	0.1111	1.8452	0.5614	2.1983	2.3823
	1	2	3	1.2927	0.1091	2.0436	0.4636	2.2075	2.2287	1.2927	0.1091	1.8440	0.5305	2.1961	2.6541
	2	3	2	2.2916	0.1100	1.9998	1.4228	2.0341	0.6237	2.2916	0.1100	1.8028	1.8835	1.8995	0.7341
	5	5	3	5.4569	0.2612	3.7844	4.4284	3.8922	4.6487	5.4569	0.2612	3.3844	5.7471	3.6419	4.8081
	9	1	1	9.1256	0.0198	0.6085	0.1607	0.6092	0.1666	9.1256	0.0198	0.5582	0.2016	0.5746	0.1944
100	0.5	1	1	0.5873	0.0097	0.6392	0.1355	0.6394	0.1382	0.5873	0.0097	0.5905	0.1731	0.6063	0.1636
	1	1	1	1.0873	0.0099	0.6309	0.1412	0.6382	0.1402	1.0873	0.0099	0.5832	0.1790	0.6016	0.1678
	2	1	1	2.0938	0.0112	0.6300	0.1423	0.6360	0.1409	2.0938	0.0112	0.5813	0.1801	0.6008	0.1673
	5	1	2	5.1123	0.0161	0.9014	0.0228	0.9253	1.2172	5.1123	0.0161	0.8180	0.0488	0.9037	1.2756
	0.5	2	3	0.7098	0.0559	2.3520	0.4855	2.4415	1.3891	0.7098	0.0559	2.0646	0.4365	2.3404	1.7400
	1	2	3	1.2126	0.0566	2.3403	0.5024	2.4421	1.3195	1.2126	0.0566	2.0487	0.4746	2.3513	1.7114
	2	3	2	2.2051	0.0527	2.3457	0.7676	2.3452	0.5990	2.2051	0.0527	2.0567	1.3122	2.1233	0.6243
	5	5	3	5.3326	0.1396	4.5322	2.7985	4.5494	5.3068	5.3326	0.1396	3.8810	4.2438	4.0326	4.5262
	9	1	1	9.0893	0.0102	0.6321	0.1407	0.6408	0.1384	9.0893	0.0102	0.5837	0.1782	0.6082	0.1622

**Table 6:** Bayesian estimates under the non-informative prior for the parameters.

	Parameters			$\hat{\mu }$		$\hat{\sigma }$		$\hat{\gamma }$	
n	μ	σ	γ	AV	MSE	AV	MSE	AV	MSE
20	0.5	1	1	0.4540	0.0146	1.0860	0.0578	1.0959	0.0537
	1	1	1	0.9663	0.0224	1.0840	0.1152	1.1168	0.0719
	2	1	1	1.9480	0.0192	1.1510	0.0817	1.0970	0.0723
	5	1	2	4.9446	0.0230	1.0487	0.0295	2.6038	1.9863
	0.5	2	3	0.4067	0.1103	2.1224	0.1448	3.6692	3.5298
	1	2	3	0.8552	0.1263	2.1850	0.1967	3.5577	1.6286
	2	3	2	1.9859	0.1003	3.5332	2.3812	2.0550	0.1575
	5	5	3	4.8681	0.5123	6.0253	12.002	3.0668	0.3777
	9	1	1	8.9256	0.0188	1.1029	0.0456	1.1759	0.0996
50	0.5	1	1	0.4797	0.0065	1.0431	0.0173	1.0386	0.0132
	1	1	1	0.9766	0.0050	1.0636	0.0171	1.0467	0.0187
	2	1	1	1.9646	0.0073	1.0537	0.0154	1.0404	0.0162
	5	1	2	4.9649	0.0118	1.0296	0.0085	2.2640	0.2529
	0.5	2	3	0.4394	0.0313	2.0580	0.0498	3.1440	0.2525
	1	2	3	0.9504	0.0338	2.0517	0.0501	3.2058	0.3199
	2	3	2	1.9347	0.0349	3.2052	0.2612	2.0955	0.0636
	5	5	3	4.8834	0.0772	5.4039	1.0454	3.1147	0.1176
	9	1	1	8.9773	0.0063	1.0398	0.0129	1.0595	0.0176
100	0.5	1	1	0.4836	0.0028	1.0243	0.0078	1.0066	0.0054
	1	1	1	0.9945	0.0030	1.0183	0.0073	1.0179	0.0095
	2	1	1	1.9811	0.0028	1.0265	0.0095	1.0207	0.0082
	5	1	2	4.9837	0.0056	1.0109	0.0056	2.0761	0.0842
	0.5	2	3	0.4729	0.0144	2.0199	0.0237	3.1091	0.1197
	1	2	3	0.9592	0.0129	2.0380	0.0220	3.1280	0.1162
	2	3	2	1.9407	0.0142	3.1002	0.0996	2.0428	0.0278
	5	5	3	4.9547	0.0406	5.2677	0.5387	3.0410	0.0565
	9	1	1	8.9854	0.0024	1.0223	0.0056	1.0287	0.0072

**Table 7:** Interval estimation results with different sample sizes.

	Parameters			μ		σ		γ	
n	μ	σ	γ	Length	Coverage	Length	Coverage	Length	Coverage
20	0.5	1	1	0.1665	0.406	0.5377	0.644	0.5385	0.642
	1	1	1	0.1672	0.440	0.5459	0.662	0.5433	0.646
	2	1	1	0.1673	0.442	0.5366	0.656	0.5368	0.628
	5	1	2	0.2107	0.436	0.4100	0.542	2.0724	0.738
	0.5	2	3	0.3935	0.452	0.8787	0.592	2.2588	0.752
	1	2	3	0.3943	0.450	0.8864	0.612	2.3670	0.702
	2	3	2	0.3931	0.442	2.1825	0.724	0.8974	0.618
	5	5	3	0.6092	0.458	4.1680	0.718	1.2952	0.550
	9	1	1	0.1673	0.432	0.5403	0.662	0.5379	0.630
50	0.5	1	1	0.1141	0.490	0.3558	0.688	0.3550	0.698
	1	1	1	0.1148	0.492	0.3568	0.706	0.3593	0.720
	2	1	1	0.1134	0.460	0.3567	0.674	0.3508	0.654
	5	1	2	0.1449	0.486	0.2772	0.626	1.2222	0.816
	0.5	2	3	0.2664	0.474	0.5918	0.594	1.3940	0.762
	1	2	3	0.2686	0.510	0.5987	0.650	1.3977	0.748
	2	3	2	0.2684	0.508	1.3988	0.730	0.5985	0.612
	5	5	3	0.4147	0.482	2.5328	0.758	0.8708	0.646
	9	1	1	0.1138	0.468	0.3522	0.682	0.3561	0.704
100	0.5	1	1	0.0841	0.496	0.2586	0.732	0.2602	0.744
	1	1	1	0.0836	0.522	0.2579	0.718	0.2577	0.726
	2	1	1	0.0834	0.480	0.2582	0.710	0.2562	0.678
	5	1	2	0.1057	0.512	0.2022	0.606	0.8376	0.796
	0.5	2	3	0.1965	0.502	0.4356	0.632	1.0005	0.778
	1	2	3	0.1966	0.498	0.4334	0.634	1.0065	0.792
	2	3	2	0.1972	0.518	1.0105	0.806	0.4346	0.624
	5	5	3	0.3035	0.456	1.8116	0.800	0.6314	0.634
	9	1	1	0.0837	0.512	0.2593	0.692	0.2571	0.700

**Table 8:** Sensitivity analysis for different loss functions (n=50).

Parameters	SELF (δ=−2)			ELF (δ=−1)			PLF (δ=1)		
	$\hat{\mu }$	$\hat{\sigma }$	$\hat{\gamma }$	$\hat{\mu }$	$\hat{\sigma }$	$\hat{\gamma }$	$\hat{\mu }$	$\hat{\sigma }$	$\hat{\gamma }$
(0.5, 1, 1)	0.4828	1.0180	1.0141	0.4827	1.0179	1.0139	0.4909	1.0258	1.0214
(5, 1, 2)	4.9705	1.0127	2.0336	4.9707	1.0123	2.0325	4.9717	1.0182	2.0672
(0.5, 2, 3)	0.4541	1.9945	2.9320	0.4536	1.9951	2.9338	0.5009	2.0077	2.9660
(5, 5, 3)	4.9072	4.9659	3.0175	4.9057	4.9678	3.0194	4.9160	5.0267	3.0372

**Table 9:** Relative efficiency for selected parameter combinations.

*n*	Parameters			Relative Efficiency (SELF/MLE)		
	μ	σ	γ	RE (μ)	RE (σ)	RE (γ)
20	0.5	1	1	0.25	0.15	0.14
	0.5	2	3	0.36	0.22	0.16
	5.0	5	3	0.70	0.22	0.35
50	0.5	1	1	0.30	0.09	0.07
	0.5	2	3	0.28	0.14	0.07
	5.0	5	3	0.27	0.08	0.17
100	0.5	1	1	0.27	0.06	0.04
	0.5	2	3	0.26	0.09	0.04
	5.0	5	3	0.29	0.05	0.12

**Table 10:** Strength data.

Strength Values									
0.562	0.564	0.729	0.802	0.950	1.053	1.111	1.115	1.194	1.208
1.216	1.247	1.256	1.271	1.277	1.305	1.313	1.348	1.390	1.429
1.474	1.490	1.503	1.520	1.522	1.524	1.551	1.551	1.609	1.632
1.632	1.676	1.684	1.685	1.728	1.740	1.761	1.764	1.785	1.804
1.816	1.824	1.836	1.879	1.883	1.892	1.898	1.934	1.947	1.976
2.020	2.023	2.050	2.059	2.068	2.071	2.098	2.130	2.204	2.262
2.317	2.334	2.340	2.346	2.378	2.483	2.683	2.835	2.835	

**Table 11:** Parameter estimates.

Distribution	MLEs	Bayes Estimates	ETT	Confidence Intervals
Rayleigh (μ,σ)	$\hat{\mu }=0.4688$	$\mu^{*}=0.2822$	ETT = 1.7955	μ:(0.0000,0.7306)
	$\hat{\sigma }=1.0585$	$\sigma^{*}=0.9095$	OBTT = 2.6248	σ:(0.100,1.2302)
Rayleigh (0,σ)	$\hat{\sigma }=1.4701$	$\sigma^{*}=1.5883$	ETT = 1.8424	σ:(1.2248,1.7153)
			OBTT = 2.6248	

**Table 12:** Fitting summary statistics for the model.

Distribution	−LogL	AIC	BIC	K–S Test	
				D Statistic	p Value
Rayleigh (μ,σ)	50.4060	104.8120	109.2803	0.2535	$2.8141\times 10^{-4}$
Rayleigh (0,σ)	57.3398	116.6795	118.9136	0.27674	$5.1408\times 10^{-5}$

## Data Availability

The original contributions presented in this study are included in the article. Further inquiries can be directed to the corresponding author.

## Notation

The following notations are used throughout this paper.

Symbol	Description
μ	Location parameter (minimum lifetime)
σ	Scale parameter for failure time distribution
γ	Scale parameter for censoring time distribution
β	$\beta =1/\sigma^{2}+1/\gamma^{2}$
α	Significance level (e.g., in confidence intervals)
$D_{i}$	Censoring indicator ($D_{i}=1$ if failure observed, 0 otherwise)
$Y_{i}$	Observed time, $Y_{i}=\min\left(X_{i},T_{i}\right)$
$w_{1}$	Number of failures, $w_{1}=\sum_{i=1}^{n}d_{i}$
$w_{2}$	$w_{2}=\sum_{i=1}^{n}{\left(y_{i}-\hat{\mu }\right)}^{2}$
